# Fingernail Onychomycosis: A Laboratory-Based Retrospective Study with Species Profiling and Antifungal Susceptibility of Yeasts

**DOI:** 10.3390/jcm15010325

**Published:** 2026-01-01

**Authors:** Paweł Krzyściak, Zuzanna Tokarz, Monika Pomorska-Wesołowska, Magdalena Skóra, Andrzej Kazimierz Jaworek, Jadwiga Wójkowska-Mach

**Affiliations:** 1 Department of Infection Control and Mycology, Chair of Microbiology, Faculty of Medicine, Jagiellonian University Medical College, Czysta 18 Street, 31-121 Kraków, Poland; zuzanna.bebenek@uj.edu.pl (Z.T.); magdalena.skora@uj.edu.pl (M.S.); 2 Department of Microbiology, Analytical and Microbiological Laboratory of KORLAB NZOZ, 41-700 Ruda Śląska, Poland; monikapw@op.pl; 3 Department of Dermatology, Faculty of Medicine, Jagiellonian University Medical College, 31-008 Kraków, Poland; andrzej.jaworek@uj.edu.pl

**Keywords:** onychomycosis, *Candida parapsilosis*, *Trichophyton*, microbial sensitivity tests, yeasts

## Abstract

**Background/Objectives:** Fingernail onychomycosis differs etiologically and epidemiologically from toenail infections and is frequently complicated by colonization and mixed growth. Reliable interpretation of microscopy–culture correlations is essential for avoiding overdiagnosis and guiding therapy. This study aimed to characterize the diagnostic structure, species distribution, and antifungal susceptibility patterns of fingernail onychomycosis in a large routine-laboratory cohort, and to evaluate the performance of a five-tier operational classification integrating microscopy and semi-quantitative culture. **Methods:** Laboratory records from 1075 patients with clinically suspected fingernail onychomycosis (including nail and periungual samples) were analyzed retrospectively (2017–2024). Direct microscopy with calcofluor white, semi-quantitative culture, and MALDI-TOF MS identification were performed. Cases were categorized based on predefined criteria combining microscopic elements with colony quantity and purity. Species distribution, age–sex patterns, diagnostic concordance between microscopy and culture, and results of EUCAST broth microdilution testing for selected yeasts were assessed. **Results:** The overall proportion of mycologically positive cases was similar in women and men, although age-dependent patterns differed. Microscopic findings correlated with culture outcomes, with hyphae predicting dermatophytes, yeast cells predicting ascomycetous yeasts, and negative slides aligning with the absence of growth. Yeasts predominated (*Candida parapsilosis* 30.9%, *C. albicans* 18.5%), dermatophytes were mainly *Trichophyton rubrum*, and molds were uncommon. Periungual swabs showed species distributions closely matching those from nail samples and demonstrated high analytical concordance. EUCAST MICs revealed species-dependent variation, including elevated amorolfine MICs in *C. parapsilosis* and reduced fluconazole activity in *Wickerhamomyces pararugosa*. **Conclusions:** Fingernail onychomycosis in this cohort was predominantly yeast-associated, with predictable microscopy–culture relationships and distinct age–sex patterns. The five-tier operational framework improved classification of infection versus colonization, and is proposed as a preliminary tool requiring clinical validation, while contemporary MIC data highlighted clinically relevant interspecies differences. The absence of clinical correlation data (symptoms, severity, treatment history) remains the primary limitation, preventing definitive distinction between infection and colonization in all cases.

## 1. Introduction

Fingernail onychomycosis is a common fungal infection of the nail apparatus that, despite being less prevalent than toenail disease, causes significant functional and aesthetic impairment [1,2]. Diagnosis is challenging because clinical features often mimic non-infectious disorders like psoriasis or trauma, necessitating laboratory confirmation [3]. While isolation of a dermatophyte is generally diagnostic [4], the significance of yeasts and non-dermatophyte molds is more equivocal, requiring careful interpretation to distinguish true infection from environmental contamination [5,6].

Unlike toenails, fingernail infections are predominantly yeast-associated with *Candida parapsilosis* emerging as a leading etiological agent, often surpassing *C. albicans* [7,8]. Factors such as mechanical or chemical trauma (e.g., cosmetic procedures) and periungual inflammation disrupt the cuticular seal and/or damage the nail barrier, creating entry points for yeasts [6,9]. Once established, these pathogens can form biofilms and produce keratinolytic enzymes [10,11,12,13,14].

The aim of this study was to delineate the diagnostic and etiological profile of fingernail onychomycosis in a large series of routine mycological investigations of clinically suspected cases. Specifically, it sought to describe the spectrum and relative frequency of fungal agents isolated from fingernails, to develop and apply a reproducible framework for interpreting combined microscopy–culture findings in terms of the likelihood of true infection, and to provide contemporary antifungal susceptibility data for yeast isolates recovered from fingernails.

## 2. Materials and Methods

Study population and data source. Data records for this study were retrieved from the electronic laboratory information system and contemporaneous handwritten bench logs of KORLAB—Rudzkie Laboratoria Medyczne (Ruda Śląska, Poland), a provider of microbiology laboratory services, including clinical mycology diagnostics, to facilities across the Silesian Voivodeship in southern Poland. The study period was 1 January 2017 to 31 December 2024. From the full database of superficial mycoses, 1075 unique patients with clinically suspected fingernail onychomycosis were identified (one diagnostic entry per patient). No records were available for May 2020 (national COVID-19 lockdown) or December 2024 (no eligible submissions). Available variables included sex, age (date of birth), direct microscopy results, culture results, and fragmentary details of lesion location. Direct microscopy was available for 1069/1075 cases (99.4%); in six cases, no preparation was performed. A periungual swab was taken in 242/1075 cases (22.5%). Laterality (right/left hand) was recorded for 350 cases, and the specific digit (I–V) for 234 cases. All records were exported, cleaned, and consolidated in a single spreadsheet (using LibreOffice Calc and LibreOffice Community, The Document Foundation, Berlin, Germany https://www.libreoffice.org, accessed 15 November 2025; Linux, Linux Foundation, San Francisco, CA, USA, https://www.linuxfoundation.org, accessed 15 November 2025).

Sample collection and diagnostic procedures. According to laboratory procedure, fingernail samples were obtained from patients with a clinical suspicion of onychomycosis, referred by dermatologists and other outpatient physicians for confirmation or exclusion of the diagnosis. Material was either collected on site at the KORLAB outpatient diagnostic laboratory or submitted from collaborating outpatient clinics and specimen collection centers. Each nail clipping was divided for direct fluorescence microscopy (10% KOH with calcofluor white, CFW) and for culture on bipartite plates containing Sabouraud dextrose agar and Fungisel™ Agar with phenol red (Graso, Starogard Gdański, Poland). Cultures were incubated for two weeks and inspected weekly; when dermatophyte growth was suspected—particularly if hyphae were observed on microscopy—incubation was extended to four weeks. All cultures were maintained at room temperature under ambient air. Species-level identification was performed by MALDI-TOF MS (Bruker Daltonics, Bremen, Germany) according to the manufacturer’s instructions.

During routine diagnostics, selected yeast isolates from fingernail samples were stored at −80 °C as part of standard laboratory practice, without systematic or consecutive collection.

Diagnostic classification of cases: To minimize overdiagnoses, we applied a weighted five-tier classification integrating microscopy with semi-quantitative culture: negative (NEG), possible positive (PssPOS), probable positive (PrbPOS), doubtful positive (DbtPOS), and proven positive (PrvPOS) (Table 1). This classification system should be considered preliminary and exploratory. While it provides a structured framework for laboratory interpretation in the absence of clinical data, its clinical validity, inter-rater reliability, and correlation with treatment outcomes have not been prospectively evaluated. The system is presented as a basis for future validation studies rather than as an established diagnostic standard.

**Interpretation of Clinical Significance:** In this analysis, different approaches were applied to interpret the clinical relevance of the obtained results. Identification of dermatophytes was considered clinically significant regardless of culture density, i.e., irrespective of the number of colony-forming units (CFU) or microscopic findings. For yeasts, an operational threshold of ≥5 CFU was adopted by the authors as the minimal indicator of potential clinical significance, including species previously classified within the genus *Candida*. This approach represents an original concept proposed by the authors, which has not been previously validated. The threshold represents an operational criterion based on our semi-quantitative culture methodology rather than a validated microbiological standard. Since nail samples were inoculated directly without standardized dilution, exact colony enumeration was not performed; the threshold was chosen to distinguish heavy growth (suggesting established colonization or infection) from sparse growth (<5 CFU, likely representing contamination). This pragmatic approach aligns with routine diagnostic mycology procedures, particularly when formal quantitative culture methods are not available, as described in international guidelines (e.g., CLSI M54). Yeast genera typically regarded as colonizers (e.g., *Rhodotorula*, *Aureobasidium*, and cutaneous cryptococci such as *Naganishia*, *Filobasidium*, and *Papillotrema*) were accepted only when microscopy confirmed yeast elements. NDMs were considered relevant solely when hyphae were documented microscopically, particularly when described as atypical.

Antifungal susceptibility testing: For this study, isolates were selected for susceptibility testing based on availability (successful recovery from frozen stocks after subculture). Temporal analysis confirmed that successfully recovered isolates were distributed relatively evenly across the study period (2017–2024). A total of 112 yeast isolates representing 19 species were recovered. For *C. parapsilosis* (*n* = 48) and *C. albicans* (*n* = 25), the tested subset is representative of the total population identified in the study. Some rare species could not be recovered due to storage or subculture failure. Antifungal susceptibility testing was performed on preserved yeast isolates using the European Committee on Antimicrobial Susceptibility Testing (EUCAST) broth microdilution method (version 7.4) [15]. The antifungal panel comprised amphotericin B, amorolfine, ciclopirox, econazole, fluconazole, itraconazole, posaconazole, terbinafine, and voriconazole (all from Pol-Aura, Poland). Isolates were revived from −80 °C stocks and subcultured on Sabouraud glucose agar (microbiological peptone 10 g/L, Oxoid, Basingstoke, UK; glucose 20 g/L, Chempur, Piekary Śląskie, Poland; agar 15 g/L, Biomaxima, Lublin, Poland). Drug dilutions were prepared in RPMI-1640 without sodium bicarbonate (Merck, Darmstadt, Germany), supplemented with 2% glucose (Chempur, Piekary Śląskie, Poland) and buffered to pH 7.0 with 0.165 M MOPS (Chemat, Konin, Poland). Growth was quantified spectrophotometrically at 530 nm after 24 h of incubation using a TECAN Infinite Pro 200 M microplate reader (Tecan, Männedorf, Switzerland). Minimum inhibitory concentrations (MICs) for azoles, amorolfine, and terbinafine were defined as the lowest concentration causing ≥50% growth inhibition relative to the drug-free control at 24 h; for amphotericin B and ciclopirox, the endpoint corresponded to ≥90% inhibition. Quality control used *Candida krusei* ATCC 6258 and *Candida parapsilosis* DSM 5784. Results were interpreted according to EUCAST clinical breakpoints for antifungal agents (version 11.0) [16].

Statistical analysis: All statistical analyses were performed in R (v4.5.0; R Foundation for Statistical Computing, Vienna, Austria) [17] using RStudio (v2025.05.1; Posit Software, PBC, Boston, MA, USA). Depending on scale and distribution, appropriate parametric or non-parametric tests were applied. Descriptive statistics comprised mean, median, interquartile range, and range. Two-sided tests with α = 0.05 were used throughout. Associations between clinical and diagnostic variables were assessed with suitable comparative procedures, and observations with missing or undefined values were excluded case-wise.

Ethical approval: The study was approved by the Bioethics Committee of the Jagiellonian University (decision no. 118.0043.1.128.2025, issued on 21 March 2025). As the study was retrospective and based solely on anonymized laboratory data, the requirement for obtaining informed consent was waived.

## 3. Results

### 3.1. Study Population and Demographics

A total of 1075 patients with suspected fingernail onychomycosis were included, comprising 744 (69.2%) females and 331 males. Among females, the median age was 58.6 years (IQR 43.5–68.2; range 0.9–96.2). Among males, the median age was 57.9 years (IQR 37.6–68.4; range 4.6–87.6). No statistically significant difference in age distribution was observed between sexes (Mann–Whitney U test, W = 127,046; *p* = 0.3024).

### 3.2. Demographic Differences Across Diagnostic Categories

The overall proportion of mycologically positive cases (POS) did not differ between females and males (43.82 vs. 43.81%; χ^2^ = 0.000, *p* = 0.997). However, when diagnostic categories were analyzed separately, distinct sex- and age-related patterns were observed (Table 2). Females were significantly overrepresented in the PssPOS group (13.8% vs. 5.7%), while males were more frequently found in the DbtPOS group (15.1% vs. 6.2%). No significant sex-based differences were noted for the proven (PrvPOS) or probable (PrbPOS) positive categories.

**Figure 1:**
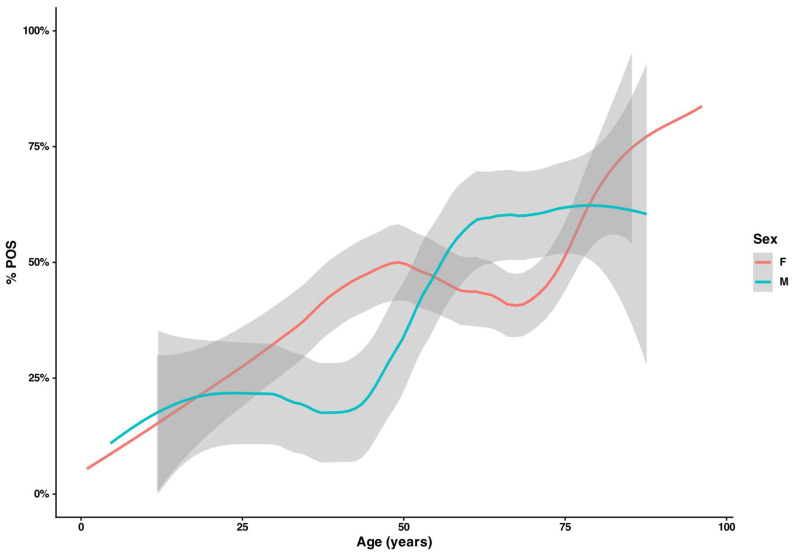
Age-specific positivity (%POS) by sex. Smoothed proportions of positive diagnoses (NEG vs. all POS) across age for women (red) and men (teal); shaded bands indicate 95% CIs. Men remain ~25% positive until the mid-40s, and then rise to ~60–65% and plateau. In women, an earlier increase to ~45–50% is observed by ~50 years of age, followed by a second upswing after ~70 years, approaching ~80% at the oldest ages; wider bands at extremes reflect sparse data.

Patients with mycologically positive results were older than those with negative findings (median 61.9 vs. 55.3 years; Mann–Whitney U, W = 108,602, *p* < 0.001). Age patterns differed by sex: among those aged 25–44 years, a higher share of women had mycological evidence of infection than men (37.1% vs. 21.9%), whereas in the 60–74-year group, males were more often affected (60.0% vs. 42.7%). Considering NEG vs. all POS, the sex-specific age curves diverged. The proportion with a mycologically positive result remains near ~25% until the mid-40s, then rises steeply to ~60–65% and stays broadly stable into late old age. In women, the probability of a positive finding increases earlier and more gradually, reaches ~45–50% by the early 50s, plateaus in midlife, and then shows a second rise after ~70 years, approaching ~80% at the oldest ages. Uncertainty increases at the extremes, as indicated by wider confidence bands (Figure 1).

Diagnostic categories, modelled with multinomial logistic regression (reference = NEG) including age, sex, and their interaction, showed a significant age × sex interaction (LR χ^2^ = 12.21, df = 4, *p* = 0.0159). With increasing age, odds for PssPOS increased in both sexes (OR per 10 years ≈1.25 in women, *p* = 0.0013; ≈1.35 in men; interaction *p* = 0.62), and odds for PrvPOS increased in both sexes but more steeply in men (≈1.25 in women, *p* = 0.0002; ≈1.69 in men; age × sex *p* = 0.007). For DbtPOS, age had no detectable effect in women (OR per 10 years ≈ 1.06, *p* = 0.54) but increased odds in men (≈1.41; age × sex *p* = 0.025). No clear age effect was observed for PrbPOS.

### 3.3. Microscopy Findings

Fungal elements were reported in 339 of 1069 available microscopy data (31.7%), including 140 with hyphae or pseudohyphae (41.3%), 40 showing mixed hyphal and yeast-like forms (11.8%), and 153 containing only yeast cells (45.1%). In six additional cases, the observed structures could not be classified. The remaining 730 samples (68.3%) were recorded as microscopy-negative.

Microscopically positive preparations were significantly more frequent in males than in females (37.2% vs. 29.3%; χ^2^ = 6.213, *p* = 0.013). No significant association was observed between hand side (left vs. right) and microscopy positivity after stratification by sex (Mantel–Haenszel χ^2^ = 0.012, *p* = 0.91; common OR = 0.98, 95% CI: 0.63–1.50).

Microscopy findings were strongly associated with culture outcome (Pearson’s chi-squared test, χ^2^(16) = 605.95, *p* < 0.001; Cramer’s V = 0.376). In slides with hyphae, dermatophytes predominated: 35.0% (49/140), and 87.5% (49/56) of all dermatophyte isolates derived from hyphae-positive specimens. When yeast cells were observed, cultures most often yielded ascomycetous yeasts: 85.0% (130/153). Microscopy-negative slides aligned mainly with no growth: 72.9% (532/730), while ascomycetes remained frequent: 22.6% (165/730) (Supplementary Materials, Table S2). A multinomial logit model (including “no growth”) corroborated these contrasts, showing higher relative risk for ascomycetes and lower relative risk for dermatophytes when slides showed yeast cells versus hyphae (RRR = 7.84, *p* < 0.001; RRR = 0.043, *p* = 0.003, respectively).

*Candida albicans* was most frequently isolated from specimens showing mixed yeast–hyphal morphology (42.5%), significantly more often than from hyphae-only (*p* < 0.001) or microscopy-negative (*p* < 0.001) samples. Yeast-only preparations also yielded *C. albicans* in 30.7% of cases, but less often than mixed forms (*p* = 0.001). By contrast, *C. parapsilosis* predominated in preparations containing only yeast cells (44.4%), which differed significantly from hyphal (*p* < 0.001) and microscopy-negative (*p* < 0.001) samples. The difference between mixed and yeast-only categories was not statistically significant (*p* = 1.00).

### 3.4. Lateralization and Digit-Specific Occurrence of Onychomycosis

The overall proportion of culture-positive results did not differ significantly between the right and left hands (57.9% vs. 51.0%; Pearson’s chi-squared test χ^2^ = 1.39, *p* = 0.240). The thumbs (Finger I) were the most frequently sampled digits and yielded the highest number of proven positive results in both hands. On the right hand, 59 of 132 cases involved the thumb, including 35 classified as proven infections. On the left hand, the thumb accounted for 43 of 102 cases, with 31 proven. The frequency of positive findings declined progressively toward the fifth finger. A similar pattern was observed for microscopy results, with hyphae detected in 20 right and 22 left thumbs.

**Figure 2:**
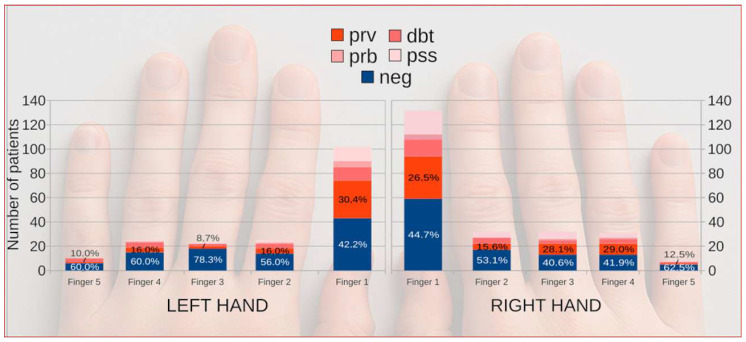
Distribution of fingernail onychomycosis across individual fingers of both hands according to diagnostic category. Colored markers indicate diagnostic certainty and microbiological confirmation (NEG, PssPOS, DbtPOS, PrbPOS, and PrvPOS). Background hand outline generated with OpenAI’s DALL·E model (2025) under permitted use; the image has no analytical meaning and serves illustrative purposes only.

When all digit-specific samples were analyzed together, the thumb accounted for approximately half of all positive cultures and showed a significantly higher positivity rate than the remaining fingers (65.1% vs. 45.8%; Pearson’s chi-squared test χ^2^ = 11.94, *p* < 0.001). The frequency of positive results declined gradually from the first to the fifth finger (Figure 2).

Species distribution followed a similar anatomical pattern. *Candida parapsilosis* and *C. albicans* predominated in the thumbs and middle digits, whereas *Trichophyton* spp. were almost exclusively recovered from thumbs and index fingers. No meaningful left–right difference was found for any taxon.

### 3.5. Etiological Agents

**Figure 3:**
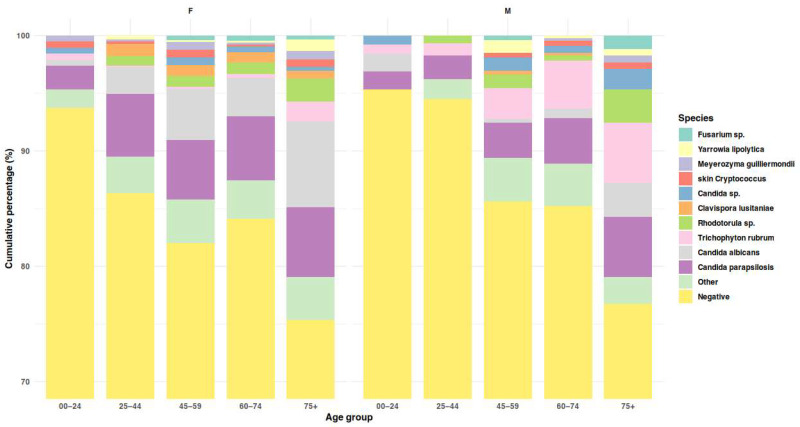
Species composition of cultured isolates across age groups, shown separately for females and males. Stacked bars display cumulative percentages of all culture outcomes (including negative results). Detailed age-related patterns are reported in the text.

Yeasts accounted for 86.1% of all isolated and identified fungal species, including ascomycetous yeasts (74.9%) and basidiomycetous yeasts (11.3%). Among yeasts, *Candida parapsilosis* predominated (approximately one-third of all isolates), followed by *C. albicans* (nearly one-fifth). Dermatophytes constituted 8.6%, all belonging to the genus *Trichophyton* (51/56 isolates were *T. rubrum*). Non-dermatophyte molds (NDMs) accounted for 5.3%, mainly *Fusarium* and *Aspergillus* species, each representing about 1.5–2% of total isolates (Table 3, Figure 3, Table S4).

Factors linked to the occurrence of the most prevalent species were analyzed using multivariable and univariable models. Among 1072 patients with complete data, *Trichophyton* was isolated in 56 cases (5.2%). Both male sex and the presence of hyphae in direct microscopy were associated with *Trichophyton* isolation (OR = 4.26, 95% CI 2.11–9.05, *p* < 0.001; OR = 52.3, 95% CI 10.9–940.4, *p* < 0.001, respectively). Age was not an independent predictor in the multivariable model; however, in univariable analysis, *Trichophyton*-positive patients were older than negatives (median 64.8 vs. 58.1 years; Mann–Whitney U: W = 20,136, *p* < 0.001). *C. parapsilosis* and *C. albicans* were detected in 18.9% and 10.2% of cases, respectively. Both species were more common in women in univariate (21.1% vs. 13.3% for *C. parapsilosis*, *p* = 0.003; 14.6% vs. 3.6% for *C. albicans, p* < 0.001; Pearson’s χ^2^) and multivariable analyses (OR for males: 0.60, 95% CI 0.40–0.88, *p* = 0.011 for *C. parapsilosis*; 0.22, 95% CI 0.11–0.41, *p* < 0.001 for *C. albicans*). Age showed a moderate but significant association with *C. albicans* isolation (OR per 10 years = 1.22, 95% CI 1.10–1.48, *p* = 0.002). For *C. parapsilosis*, the age effect was borderline and small (OR per 10 years = 1.10, 95% CI 1.00–1.22, *p* = 0.053). In univariable analyses, patients positive for *C. parapsilosis* or *C. albicans* were older than negatives (Mann–Whitney U: 58.7 vs. 55.0 years, *p* = 0.009; 62.2 vs. 55.1 years, *p* < 0.001).

The relative frequencies of all major taxa remained stable over time. Minor year-to-year variations, including a slight decrease in *Trichophyton* spp., were not statistically significant (*p* = 0.14; Supplementary Materials, Figure S1).

### 3.6. Analysis of Periungual Sampling Records

A total of 123/242 (50.8%) periungual swabs yielded fungal growth. The distribution of outcomes differed significantly across diagnostic categories (χ^2^ = 107.2, df = 12, *p* < 0.001). Positive swabs ≥ 5 CFU were found in 67.8% of PrvPOS and 63.0% of PssPOS mycoses, whereas only 15.3% of mycologically negative cases yielded fungal growth. Intermediate results predominated among PrbPOS cases (50.0%), while negative cultures prevailed among negative cases (88.5%). In the doubtful category (DbtPOS), periungual swabs were predominantly negative (42.4%) or showed only minimal growth (15.1%) (Supplementary Materials, Table S3).

The most frequent isolates were *C. parapsilosis* (45.9%) and *C. albicans* (19.7%), followed by *Candida* sp. not further identified (7.0%), *Rhodotorula* sp. (5.7%), *Clavispora lusitaniae* (3.8%), and *Meyerozyma guilliermondii* (2.5%). Other yeasts, including *C. orthopsilosis*, *C. tropicalis*, *Nakaseomyces glabratus*, and *Cryptococcus* sp., each accounted for ≤2% of samples.

Comparison with nail plate cultures demonstrated an overall agreement of 69.0% and moderate concordance (Cohen’s κ = 0.38). Among 89 double-positive cases, at least one identical species was isolated from both specimens in 85 (95.5%). Multi-species infections were observed in 17 of 89 (19.1%) double-positive pairs, most frequently involving combinations of *C. albicans* and *C. parapsilosis*, occasionally together with *C. lusitaniae* or *M. guilliermondii*.

### 3.7. Yeasts Antifungal Susceptibility

Table 4 summarizes MIC distributions (geometric mean, range; MIC_50_/MIC_90_) for nine antifungals tested against five predominant yeast species isolated from fingernails. Overall, triazoles showed low MICs—especially posaconazole and voriconazole—while fluconazole exhibited reduced activity against *W. pararugosa* (high GM, MIC_50_/_90_ = 8/8 mg/L). Terbinafine activity was species-dependent (i.e., very low MICs for *C. parapsilosis*, higher for *C. albicans*) (Table 4, Supplementary Materials Figure S3).

Among remain rare yeasts (*n* = 20), eight isolates showed high terbinafine MICs (>1 mg/L), including *Naganishia diffluens* (2), *Rhodotorula* sp., *Yarrowia lipolytica*, *Candida tropicalis* (2), *Kazachstania* sp., and *Kluyveromyces marxianus*. Very high fluconazole MICs were recorded for basidiomycetous yeasts (*N. diffluens*, *Rhodotorula* sp.; 64 mg/L) and for C. sake (8 mg/L). Despite these findings, all rare yeasts remained inhibited by amphotericin B at low MICs (≤1 mg/L). Detailed MIC values are presented in Supplementary Materials Table S1. 

## 4. Discussion

We developed and proposed a preliminary five-tier classification system as an exploratory framework for laboratory interpretation that integrates the presence and morphology of fungal elements in direct microscopy with the extent and purity of culture growth. This structured framework was designed to compensate for the lack of clinical data, providing a more objective basis for interpreting purely laboratory findings. The approach, intentionally weighted toward specificity, aims to minimize overdiagnosis while maintaining reproducibility under routine conditions. The concept is analogous to evidence-based categorical systems used in invasive fungal disease diagnostics [18] and may serve as a basis for more reliable evaluation once supported by clinical correlation (e.g., dermoscopy), particularly as diagnosis increasingly shifts toward DNA-based methods. The five-tier classification was intentionally designed to be compatible with future clinical integration. In prospective studies, categories such as PssPOS and DbtPOS could trigger standardized clinical assessment protocols (including dermoscopy, patient history of trauma or immunosuppression, and previous treatment), whereas PrvPOS cases might proceed directly to therapy. Validation studies correlating each tier with clinical outcomes and treatment response rates are needed to establish the system’s diagnostic accuracy and clinical utility. Although the ≥5 CFU threshold for yeasts is an operational rather than an absolute criterion, it provides a practical gauge of potential clinical significance in the absence of quantitative inoculation. Future studies incorporating histopathology or molecular assays could further refine these thresholds.

In this study, the overall proportion of mycologically positive versus negative results did not differ significantly between women and men, in contrast to reports suggesting higher rates of fingernail onychomycosis in women [19,20,21]. This discrepancy likely reflects methodological differences. First, the laboratory routinely used calcofluor white (CFW) fluorescence, which is more sensitive than potassium hydroxide (KOH) wet mounts for detecting fungal elements, particularly yeast cells [22,23,24]. Second, we applied explicit case definitions supported by microscopy (presence of yeast cells and/or hyphae) together with a colony-count threshold. Microscopy findings differed markedly by sex. Hyphae—suggestive of dermatophytes—were recorded far more often in specimens from men than from women (82/331, 24.8% vs. 58/744, 7.8%), whereas yeast and mixed elements were more frequent in women (153/744, 20.6% vs. 40/331, 12.1%), consistent with prior data. The observed predominance of yeasts in women’s fingernails and of dermatophytes in men’s fingernails warrants dedicated studies; plausible contributors include sex-related differences in nail-plate keratin composition [25], hormonal influences [26], and differential exposure (behavioral factors).

However, age-stratified analysis revealed distinct, non-linear, sex-dependent trends. In a spline logistic regression, the main effect of sex was not significant, but the age × sex interaction was (*p* = 0.007). In men, the late-onset increase is consistent with evidence that male sex, cumulative nail trauma, toenail onychomycosis, and comorbidities such as diabetes and peripheral vascular disease are major risk factors for onychomycosis, particularly dermatophyte infections. In women, the age–positivity curve was biphasic, with a prolonged plateau around 40% between 45 and 70 years; the early rise aligns with reports that candidal fingernail onychomycosis is more common in younger and middle-aged women, often associated with wet-work exposure and the growing use of cosmetic nail procedures (manicure, artificial nails). The late-life upswing in both sexes reflects the well-recognized impact of advanced age—reduced nail growth, vascular insufficiency, polypharmacy and functional decline—so that from ≥75 years the predicted probabilities converge and sex-specific differences largely disappear (Figure 1). Species profiles also differed: men were more frequently affected by dermatophytes, whereas women more often yielded yeasts. Taken together, these findings indicate that sex per se is not a primary determinant of fungal positivity, but its interaction with age strongly shapes diagnostic distributions, producing contrasting patterns in younger women and older men.

Right-hand predominance in fingernail onychomycosis has often been reported [27,28]. In our cohort, although right-sided involvement prevailed, lateralization was not statistically significant. This suggests that hand dominance may play a limited role compared with local mechanical stress.

According to Iorizzo et al. [27], the second and third fingers were most frequently affected, whereas Papini et al. observed thumb involvement in 44.4% of cases, with multiple-digit lesions in 56% [28]. In our study, information on the specific digit examined was available for 339 patients; among these, the thumb was most frequently affected (209/339; 61.7%). This pattern likely reflects the thumb’s predominant mechanical and functional exposure—including frequent contact with contaminated surfaces, microtrauma, and humidity accumulation during manual tasks. Multiple-digit involvement was recorded in 39 patients (11.5%), while a single digit was affected in 300 cases. However, these proportions should be interpreted cautiously, as for more than 700 patients, the exact sampling site was not specified in the records.

Paronychia is an inflammatory disorder of the proximal and lateral nail folds that may result from bacterial, *Candida*, or viral infections, but can also mimic non-infectious conditions, including contact dermatitis, psoriasis, trauma-related changes, or neoplasia such as squamous cell carcinoma, necessitating careful differential diagnosis [29].

In our study, periungual swabs were collected from 242/1075 patients (22.5%), with fungal growth in 123/242 (50.8%). These figures are comparable to prior reports, where chronic paronychia accompanied candidal onychomycosis in 35% of cases (35/101) [30], and culture positivity reached 56.1% [31]. However, some studies question the etiological role of *Candida* in paronychia and suggest that it may often represent secondary colonization rather than primary infection [9].

Similarly to nail plate cultures, the predominant species in periungual samples was *C. parapsilosis* (45.9%), followed by *C. albicans* (19.7%), together accounting for two-thirds of all isolates. This differs from other studies showing predominance of *C. albicans* [31,32]. The high rate of species-level concordance between periungual and nail isolates (95.5%), with a balanced distribution of discordant results (McNemar test, *p* = 0.49), confirms that both specimen types detect largely the same etiologic agents and the continuum of pathological process. Discordant pairs most commonly involved environmental or opportunistic yeasts (e.g., *Rhodotorula* spp., unidentified *Candida* spp.), which likely represent transient colonization or superficial contamination rather than true etiologic discrepancies.

Advances in diagnostic methodology have reshaped the observed species distribution in fingernail onychomycosis by improving recognition of yeasts previously misidentified with culture-based techniques [33]. In our material, *C. parapsilosis* sensu stricto was the most frequently isolated species (31% of all isolates), exceeding *C. albicans* (18.5%). Similar predominance of *C. parapsilosis* has been reported elsewhere [7,34,35,36], although some settings still show *C. albicans* dominance [37,38]. Although *C. parapsilosis* was most frequent overall, *C. albicans* displayed stronger pathogenic indicators: it more often coincided with supportive microscopy (hyphae/pseudohyphae), was the sole or dominant isolate in “proven” episodes, and had documented invasive potential in nails [39,40]. In contrast, *C. parapsilosis* frequently co-occurred with other yeasts. Co-isolation with *C. albicans* occurred in 32 multi-isolate episodes, about half of which met “proven” criteria (54.5%), indicating that co-recovery may reflect infection but does not by itself establish dual causality. Cryptic members of the *C. parapsilosis* complex were rare and did not influence the overall pattern: *C. orthopsilosis* and *C. metapsilosis* together accounted for <10% of *C. parapsilosis* sensu lato, in line with Polish data [41].

Dermatophytes constituted 8.6% of all isolates, mainly *Trichophyton rubrum* (7.9%), consistent with a U.S. laboratory report (7.8%) [42] and higher than a Polish series from Gdańsk (3.3% of culture-positive samples) [43]. When microscopy-positive, culture-negative preparations with true hyphae were counted as presumed dermatophyte infections, the proportion increased to 13%, approaching the figures of Gawdzik et al. (16.6% dermatophytes; 75.5% *Candida* spp.) [44].

Basidiomycetous yeasts formed the fourth most common group (*Rhodotorula* spp. 6.48%; cutaneous cryptococci 3.7%). A comparable prevalence of *Rhodotorula* (3.1%) in fingernails was reported by Gawdzik et al. [44]. Although basidiomycetous yeasts are generally regarded as colonizers, isolated reports have shown *Rhodotorula* meeting strict aetiologic criteria in selected nail infections [45]. In our data, among the 18 episodes where *Rhodotorula* or cutaneous cryptococci were the sole isolates, only one met the “proven” category (supportive microscopy with abundant growth). In four additional episodes, microscopy showed true hyphae, suggesting an undetected dermatophyte rather than yeasts. The remainder lacked supportive microscopy and were interpreted as colonization.

Uncommon ascomycetous yeasts were a distinct minority yet not negligible: *Clavispora lusitaniae* (4.2%), *Meyerozyma guilliermondii* (2.0%), *Yarrowia lipolytica* (2.0%), and *Wickerhamomyces pararugosa* (1.5%) were each detected in small proportions, broadly consistent with other laboratory-based reports [46,47,48]. *Pichia kudriavzevii* (formerly *Candida krusei*) and *Nakaseomyces glabratus* (formerly *Candida glabrata*) were extremely rare in our material (<0.5% of all isolates; 0.6% among ascomycetous yeasts), whereas some series list them among more frequent non-C. *albicans* yeasts in fingernails [49], indicating substantial regional and methodological variation. These species are generally low-virulence colonizers but may act as opportunistic pathogens when repeatedly isolated with supportive microscopy. Environmental and cosmetic exposures may shape this “long-tail” composition; in line with Hedderick et al. [50], artificial nails have been associated with higher recovery of non-C. *albicans* yeasts, including *C. parapsilosis* and *Y. lipolytica*. Although exposure data were unavailable in our records, female predominance and age-stratified patterns support a plausible role for nail cosmetics and material interfaces. Some rare yeasts also show reduced antifungal susceptibility, potentially facilitating persistence after prior therapy.

Collectively, the data support a pathogenicity gradient: dermatophytes—particularly *T. rubrum*—and *C. albicans* act as primary (“true”) nail pathogens, *C. parapsilosis* functions more often as a facultative/secondary pathogen, and basidiomycetous yeasts predominantly represent colonization.

There remains a marked paucity of antifungal susceptibility data for yeasts isolated from fingernails. The study by Figueiredo et al. [7] provided important reference MIC values using both the Clinical and Laboratory Standards Institute (CLSI) and EUCAST methods for 200 strains for common species (*C. albicans*, *C. parapsilosis*, and *C. tropiclais*) isolated from finger nails. Our dataset contributes further evidence by offering a contemporary EUCAST MIC collection for yeast-associated fingernail onychomycosis (*C. parapsilosis*, *C. albicans*), including several uncommon taxa rarely evaluated in this setting (i.e., *M. guilliermondii*).

Our susceptibility data reinforce current treatment guidelines while highlighting important species-specific considerations. Current Polish and international guidelines [6,51] recommend topical therapy (amorolfine, ciclopirox) or systemic azoles (fluconazole, itraconazole) for yeast onychomycosis. While EUCAST clinical breakpoints are primarily established for systemic infections, they provide reasonable guidance for oral therapy in onychomycosis, particularly for itraconazole, which has well-documented nail penetration. Our data show that terbinafine MICs for *C. parapsilosis* are substantially lower than for *C. albicans* (MIC_90_ = 0.125 vs. 5.6 mg/L), supporting guideline recommendations for considering systemic terbinafine specifically for *C. parapsilosis* onychomycosis. Notably, *C. parapsilosis* showed markedly higher amorolfine MICs (MIC_90_ = 4.0 mg/L), which may explain some topical treatment failures and could support preferential use of alternative topical agents or systemic therapy for this species. 

Moreover, yeasts outside the major pathogenic *Candida* species frequently displayed high MICs to standard topical and systemic agents, explaining why basidiomycetous yeasts and other low-susceptibility taxa may be recovered during or after therapy directed against dermatophytes—either obscuring the true etiology when present as colonizers or delaying normalization of the nail when possessing keratinolytic potential, as reported for *Rhodotorula mucilaginosa* [52].

For topical agents (amorolfine, ciclopirox), no EUCAST breakpoints exist, as interpretive criteria for topical therapy are not defined. This represents an important gap in antifungal stewardship for superficial infections. Our MIC data may contribute to future discussions on establishing nail infection-specific interpretive criteria. Furthermore, the five-tier diagnostic classification proposed in this study could provide a standardized framework for correlating MIC values with clinical outcomes in future prospective studies, potentially enabling the development of evidence-based breakpoints for onychomycosis.

Our results confirm that azoles remain highly effective against major pathogens, with MIC_90_ values well below breakpoints. However, three species-specific patterns emerge that merit particular attention. First, *C. parapsilosis* exhibited notably lower terbinafine MICs (MIC_90_ = 0.125 mg/L) compared to *C. albicans* (MIC_90_ = 5.6 mg/L), a finding that supports guideline recommendations for considering systemic terbinafine specifically for *C. parapsilosis* infections. In contrast, this species showed elevated amorolfine MICs (MIC_90_ = 4.0 mg/L), which may explain some topical treatment failures and suggests that alternative topical therapies might be preferable. Finally, high fluconazole MICs detected in *W. pararugosa* and reduced susceptibility in basidiomycetous yeasts (e.g., *Rhodotorula*) emphasize the value of species-level identification in refractory cases. These organisms may persist during dermatophyte-targeted therapy, potentially acting as resistant colonizers or delaying cure (Table 4).

### Limitations

The principal limitation of this study is the lack of clinical correlation data, including patient symptoms, disease severity, treatment history, and clinical outcomes. This prevents definitive determination of whether isolated organisms represent true pathogens, secondary colonizers, or contaminants in individual cases. The five-tier classification was designed to partially compensate for this limitation by integrating multiple laboratory parameters, but clinical validation remains essential. Additionally, the diagnostic protocol relied on phenotypic methods (culture and MALDI-TOF MS) without routine molecular detection directly from nail tissue (PCR). Given that dermatophytes can be difficult to culture, their prevalence might be underestimated compared to studies using molecular techniques. Furthermore, isolates for antifungal susceptibility testing were retrieved from frozen stocks based on availability; this “survivorship bias” means the tested subset might not fully reflect the susceptibility profile of fragile or fastidious strains that failed to recover after thawing. Finally, as data originate from a single laboratory serving a specific region in southern Poland, the observed species distribution may reflect local epidemiological factors and referral patterns. Future prospective studies should systematically collect clinical metadata, including nail involvement score, patient risk factors, dermoscopy findings, and treatment responses, to establish the clinical validity of laboratory-based diagnostic criteria.

## 5. Conclusions

This retrospective laboratory-based study of 1075 fingernail samples confirms that fingernail onychomycosis is predominantly a yeast-associated disease, characterized by the emergence of *Candida parapsilosis sensu stricto* as the leading pathogen and distinct age–sex epidemiological patterns. The proposed five-tier operational classification offers a standardized framework for distinguishing infection from colonization in routine diagnostics, mitigating the challenges posed by the absence of clinical data. Furthermore, antifungal susceptibility testing revealed clinically relevant species-specific profiles—notably lower terbinafine MICs but elevated amorolfine MICs in *C. parapsilosis* compared to *C. albicans*—which may necessitate adjustments in therapeutic strategies. Despite the limitation of lacking clinical correlation, these findings provide a robust evidentiary basis for updating diagnostic algorithms and interpreting microbiological results in dermatological practice.

## Figures and Tables

**Table 1:** Rationale of the five-tier classification of fingernail onychomycosis. The system categorizes cases based on microscopy findings, culture results, and colony counts, including species-specific thresholds for diagnostic relevance.

Category	Microscopy Criterion	Culture Criterion	Rationale (Clinical Mycology)
NEG (Negative)	No fungal elements	<5 CFU on media	Both primary tests are negative, so there is no laboratory evidence of onychomycosis.
PssPOS (Possible +)	No fungal elements	≥5 CFU of a clinically relevant yeast (species formerly classified in the *Candida* genus, e.g., *C. parapsilosis*, *C. albicans*)	A sizeable yeast load without microscopic confirmation may represent an early or focal infection; dermatological correlation and/or repeat culture are recommended.
DbtPOS (Doubtful +)	Fungal elements present (yeast cells or hyphae)	(i) No growth of clinically relevant fungi and only scant growth (<5 CFU) of common contaminants; or (ii) non-correspondence between microscopy and culture (e.g., yeast cells in microscopy with dermatophyte growth, or abundant hyphae in microscopy while a yeast species that does not produce hyphae/pseudohyphae is isolated)	Fungal structures are seen, yet culture is absent or discordant—may reflect non-viable organisms (prior therapy, desiccation) or suboptimal culture; the result is inconclusive.
PrbPOS (Probable +)	Fungal elements morphologically consistent with the culture isolate	<5 CFU of a clinically relevant yeast (i), or ≥5 CFU of a colonizing yeast (e.g., *Rhodotorula*, *Naganishia*) (ii)	(i) Microscopy shows fungal elements but only scant growth occurs—typical of treated or small samples. (ii) Alternatively, abundant growth of yeasts usually regarded as colonizers may indicate an emerging infection. Both scenarios suggest infection but do not prove it.
PrvPOS (Proven +)	Yeast cells, hyphal elements, or atypical fungal structures consistent with the cultured organism; may be negative in dermatophyte infections	(i) ≥5 CFU of a yeast species; or (ii) any growth of *Trichophyton* spp.; or (iii) growth of a non-dermatophytic mold matching the atypical microscopy	Full concordance between tests (i), isolation of a recognized primary pathogen (ii), or consistent mold morphology when no previous treatment (iii) establishes etiological certainty.

CFU—colony-forming units.

**Table 2:** Sex-specific distribution of diagnostic categories and results of Pearson’s chi-squared tests (with Yates’ correction) comparing the frequency of each category (POS, PrvPOS, PrbPOS, DbtPOS, PssPOS, NEG) between males and females. The *p*-values indicate statistical significance for certain diagnostic categories. NA data are omitted.

Case Category	0–24		25–44		45–59		60–74		75+		Total	
	F	M	F	M	F	M	F	M	F	M	F	M
Total POS	9 (18.8%)	6 (18.8%)	53 (37.1%)	16 (21.9%)	86 (46.5%)	27 (40.9%)	125 (42.7%)	69 (60.0%)	47 (63.5%)	26 (60.5%)	326 (43.8%)	145 (43.8%)
PrvPOS ^1^	4 (8.3%)	1 (3.1%)	22 (15.4%)	6 (8.2%)	38 (20.5%)	12 (18.2%)	66 (22.5%)	33 (28.7%)	22 (29.7%)	17 (39.5%)	152 (20.4%)	69 (20.8%)
PrbPOS ^2^	0 (0.0%)	1 (3.1%)	3 (2.1%)	2 (2.7%)	7 (3.8%)	1 (1.5%)	7 (2.4%)	3 (2.6%)	2 (2.7%)	0 (0.0%)	19 (2.5%)	7 (2.1%)
DbtPOS ^3^	3 (6.2%)	3 (9.4%)	12 (8.4%)	7 (9.6%)	10 (5.4%)	7 (10.6%)	16 (5.5%)	25 (21.7%)	5 (6.8%)	7 (16.3%)	46 (6.2%)	50 (15.1%)
PssPOS ^4^	2 (4.2%)	1 (3.1%)	16 (11.2%)	1 (1.4%)	31 (16.8%)	7 (10.6%)	36 (12.3%)	8 (7.0%)	18 (24.3%)	2 (4.7%)	103 (13.8%)	19 (5.7%)
NEG	38 (79.2%)	26 (81.2%)	89 (62.2%)	57 (78.1%)	98 (53.0%)	39 (59.1%)	165 (56.3%)	46 (40.0%)	27 (36.5%)	17 (39.5%)	418 (56.2%)	186 (56.2%)
POS vs. NEG statistics	χ^2^ = 0.002 *p* =0.965		χ^2^ = 5.251 *p* = 0.022		χ^2^ = 0.667 *p* = 0.414		χ^2^ = 9.420 *p* =0.002		χ^2^ = 0.108 *p* = 0.743		χ^2^ = 0.000 *p* = 0.997	

Statistics for total numbers in each POS category vs. NEG: ^1^—χ^2^ = 0.024, *p* = 0.876; ^2^—χ^2^ = 0.187, *p* = 0.665; ^3^—χ^2^ = 22.427, *p* < 0.001; ^4^—χ^2^ = 14.954, *p* < 0.001.

**Table 3:** Summary of the predominant fungal species identified in fingernail onychomycosis cases. Only species with a prevalence >1% are shown individually. For the complete species profile, including rare isolates, see Table S1 in the Supplementary Materials.

Group of Fungi		Genus	Species	Number	Percentage
YEAST	ASCOMYCETOUS	*CANDIDA*	*Candida albicans* complex	122	18.83
			*Candida parapsilosis* complex	208	32.10
			*Candida tropicalis*	11	1.70
			subtotal CANDIDA	341	52.62
		OTHER SPECIES	*Clavispora lusitaniae*	27	4.17
			*Debaryomyces hansenii*	7	1.08
			*Geotrichum candidum*	8	1.23
			*Hanseniospora* spp.	11	1.70
			*Meyerozyma guilliermondii*	13	2.00
			*Sungouiella intermedia*	8	1.23
			*Wickerhamiella pararugosa*	10	1.54
			*Yarrowia lipolytica*	13	2.00
			other	18	3.68
			subtotal other yeast	117	18.06
			*Candida sp.* (not species ID)	23	3.55
		subtotal ascomycetous yeasts		485	74.85
	BASIDIOMYCETOUS	*RHODOTORULA*	*Rhodotorula* spp.	43	6.64
		SKIN CRYTPOCOCCI	*Filobasidium, Naganishia, Papillotrema*	24	3.7
		OTHER SPECIES	*Trichosporon* spp., *Aureobasidium* spp.	2	0.92
		subtotal basidiomycetous yeasts		73	11.27
	subtotal YEASTS			558	86.12
DERMATOPHYTES			other *Trichophyton*	5	0.77
		*TRICHOPHYTON*	*Trichophyton rubrum*	51	7.87
		subtotal DERMATOPHYTES		56	8.64
MOLDS		*ASPERGILLUS*	*Aspergillus* sp.	10	1.54
		*FUSARIUM*	*Fusarium* spp.	13	2.01
		OTHER GENERA		11	1.70
		subtotal MOLDS		34	5.25
TOTAL				648	100

**Table 4:** Antifungal susceptibility of the five most frequent yeast species. Values represent geometric mean MICs (GM) with range, and MIC_50_/MIC_90_ values [mg/L] determined by the EUCAST broth microdilution method (v. 7.4).

Antifungal Agent (µg/mL)		*C. parapsilosis* (*n* = 48)	*C. albicans* (*n* = 25)	*C. lusitaniae* (*n* = 9)	*M. guilliermondii* (*n* = 5)	*W. pararugosa* (*n* = 5)
Econazole	GM (range)	0.250 (0.016–2.000)	0.051 (0.016–2.000)	0.068 (0.016–1.000)	0.379 (0.125–1.000)	0.758 (0.500–2.000)
	MIC_50_/MIC_90_	0.250/1.000	0.016/1.400	0.031/1.000	0.250/1.000	0.500/2.000
Fluconazole	GM (range)	0.459 (0.031–1.000)	0.244 (0.016–16.000)	0.429 (0.125–16.000)	0.500 (0.125–8.000)	5.278 (1.000–8.000)
	MIC_50_/MIC_90_	0.500/1.000	0.125/3.800	0.250/16.000	0.250/8.000	8.000/8.000
	CBP/ECOFF	4/2	4/0.5	nd/nd	nd/(16)	nd/nd
Itraconazole	GM (range)	0.043 (0.008–0.500)	0.018 (0.008–1.000)	0.047 (0.008–0.250)	0.125 (0.031–1.000)	0.250 (0.125–0.500)
	MIC_50_/MIC_90_	0.031/0.313	0.016/0.119	0.031/0.250	0.063/1.000	0.250/0.500
	CBP	0.125	0.06/0.03	nd/0.125	nd/(1)	nd/nd
Posaconazole	GM (range)	0.009 (0.008–0.063)	0.011 (0.008–1.000)	0.008 (0.008–0.008)	0.024 (0.008–0.063)	0.072 (0.031–0.125)
	MIC_50_/MIC_90_	0.008/0.016	0.008/0.044	0.008/0.008	0.031/0.063	0.063/0.125
	CBP	0.06/0.06	0.06/0.06	nd/nd	nd/0.25	nd/nd
Voriconazole	GM (range)	0.016 (0.008–0.125)	0.017 (0.008–0.250)	0.014 (0.008–0.125)	0.041 (0.008–0.125)	0.189 (0.125–0.500)
	MIC_50_/MIC_90_	0.016/0.031	0.008/0.088	0.008/0.125	0.031/0.125	0.125/0.500
	BP	0.25/0.06	0.25/0.03	nd/nd	nd/nd	nd/nd
Terbinafine	GM (range)	0.028 (0.008–8.000)	0.608 (0.016–8.000)	0.184 (0.016–1.000)	0.110 (0.016–2.000)	0.250 (0.125–1.000)
	MIC_50_/MIC_90_	0.016/0.125	1.000/5.600	0.250/1.000	0.063/2.000	0.250/1.000
Amorolfine	GM (range)	0.698 (0.016–8.000)	0.109 (0.008–2.000)	0.250 (0.125–1.000)	0.574 (0.250–1.000)	0.501 (0.063–8.000)
	MIC_50_/MIC_90_	1.000/4.000	0.125/1.400	0.125/1.000	0.500/1.000	0.500/8.000
Ciclopirox	GM (range)	0.515 (0.250–1.000)	0.473 (0.250–0.500)	0.500 (0.500–0.500)	0.500 (0.500–0.500)	0.574 (0.500–1.000)
	MIC_50_/MIC_90_	0.500/0.500	0.500/0.500	0.500/0.500	0.500/0.500	0.500/1.000
Amphotericin B	GM (range)	0.199 (0.063–1.000)	0.179 (0.031–1.000)	0.107 (0.063–0.250)	0.190 (0.063–1.000)	0.250 (0.250–0.250)
	MIC_50_/MIC_90_	0.250/0.275	0.125/0.700	0.125/0.250	0.125/1.000	0.250/0.250
	CBP	1.0/1.0	1.0/1.0	nd/(0.5)	nd/(0.5)	nd/nd

GM—geometric mean; MIC50/MIC90—minimum inhibitory concentration required to inhibit 50%/90% of isolates. CBP—EUCAST clinical breakpoint v.12; ECOFF—epidemiological cutoff value; in parentheses (x), tentative ECOFFs are indicated; nd—not defined.

## Data Availability

The data presented in this study are available upon reasonable request from the first author. The data are not publicly accessible due to institutional privacy and data protection regulations.

## Supplementary Materials

The following supporting information can be downloaded at: https://www.mdpi.com/article/10.3390/jcm15010325/s1, Figure S1: Bimonthly distribution of selected fungal species isolated from fingernails. Bars show the percentage of positive samples in each 2-month period, the black line represents a binomial generalized linear model (binomial GLM) fit weighted by the total number of samples, and semi-transparent bars indicate periods with fewer than 10 samples. Figure S2: Heatmap showing direct microscopy findings across major fungal groups identified in culture. Percentages are based on all samples in each microscopy-by-sex group; a single sample can contribute to more than one fungal group if multiple organisms are present. Figure S3: MIC distributions for fungal species–drug combinations with at least five isolates. Each panel shows the distribution of minimum inhibitory concentration (MIC) values for a specific species–antifungal pair. Bars represent the number of isolates at each observed MIC value (mg/L). Table S1: Minimal inhibitory concentration (MIC) values (mg/L) for yeasts represented by small numbers in the study material. Table S2: Concordance between direct microscopic evaluation of fingernail samples and culture results. The table shows the number of samples by isolated fungal groups (dermatophytes, ascomycetous yeasts, basidiomycetous yeasts, non-dermatophyte moulds, culture-negative) and by microscopy findings. Table S3: Distribution of periungual swab outcomes across operational diagnostic categories. Swab outcomes were classified as *swab negative* (no growth), *swab intermediate* (1–4 colonies), *swab positive concordant* (growth in both swab and nail samples), and *swab positive discordant* (growth detected in the swab only). Percentages are shown within each swab category across diagnostic groups (NEG, DbtPOS, PrbPOS, PssPOS, PrvPOS; see Table 1 in the main text). Swab outcomes were significantly associated with diagnostic classification (χ^2^, *p* < 0.001). Table S4: Comprehensive distribution and frequency of all fungal species identified in fingernail onychomycosis.

## Abbreviations

The following abbreviations are used in this manuscript:

AMB	Amphotericin B
AMO	Amorolfine
ATCC	American Type Culture Collection
BP	Break point
CFU	Colony-forming unit
CFW	Calcofluor white
CI	Confidence interval
CLSI	Clinical and Laboratory Standards Institute
CPX	Ciclopirox
DbtPOS	Doubtful positive
DSM	Deutsche Sammlung von Mikroorganismen
ECO	Econazole
EUCAST	European Committee on Antimicrobial Susceptibility Testing
FLU	Fluconazole
GM	Geometric mean
ID	Identification
ITR	Itraconazole
ITS	Internal transcribed spacer region
KOH	Potassium hydroxide
MALDI-TOF	Matrix-assisted laser desorption/ionization time-of-flight
MIC, MIC_50_, MIC_90_	Minimum inhibitory concentration, MIC inhibiting 50%/90% of isolates
NDM	Non-dermatophyte mold(s)
NEG	Negative (no evidence of onychomycosis)
OR	Odds ratio
POS	Posaconazole
PrbPOS	Probable positive
PrvPOS	Proven positive
PssPOS	Possible positive
RPMI-1640/RPMI	Roswell Park Memorial Institute 1640 medium
TER	Terbinafine
VOR	Voriconazole
