# Supplementary material for: Fingernail Onychomycosis: A Laboratory-Based Retrospective Study with Species Profiling and Antifungal Susceptibility of Yeasts
Source: J Clin Med. 2026 Jan 1;15(1):325. doi: 10.3390/jcm15010325 (PMC12787257; doi:10.3390/jcm15010325)
Supplement: Supplementary file 1 [file jcm-15-00325-s001.zip › jcm-4040635-supplementary.pdf]

Fingernail onychomycosis: a laboratory-based retrospective study with species profiling and antifungal susceptibility of yeasts

Paweł Krzyściak [1*], Zuzanna Tokarz [2], Monika Pomorska-Wesołowska [3], Magdalena Skóra [4], Andrzej Jaworek [5] and Jadwiga Wójkowska-Mach [6,*]

Supplementary material

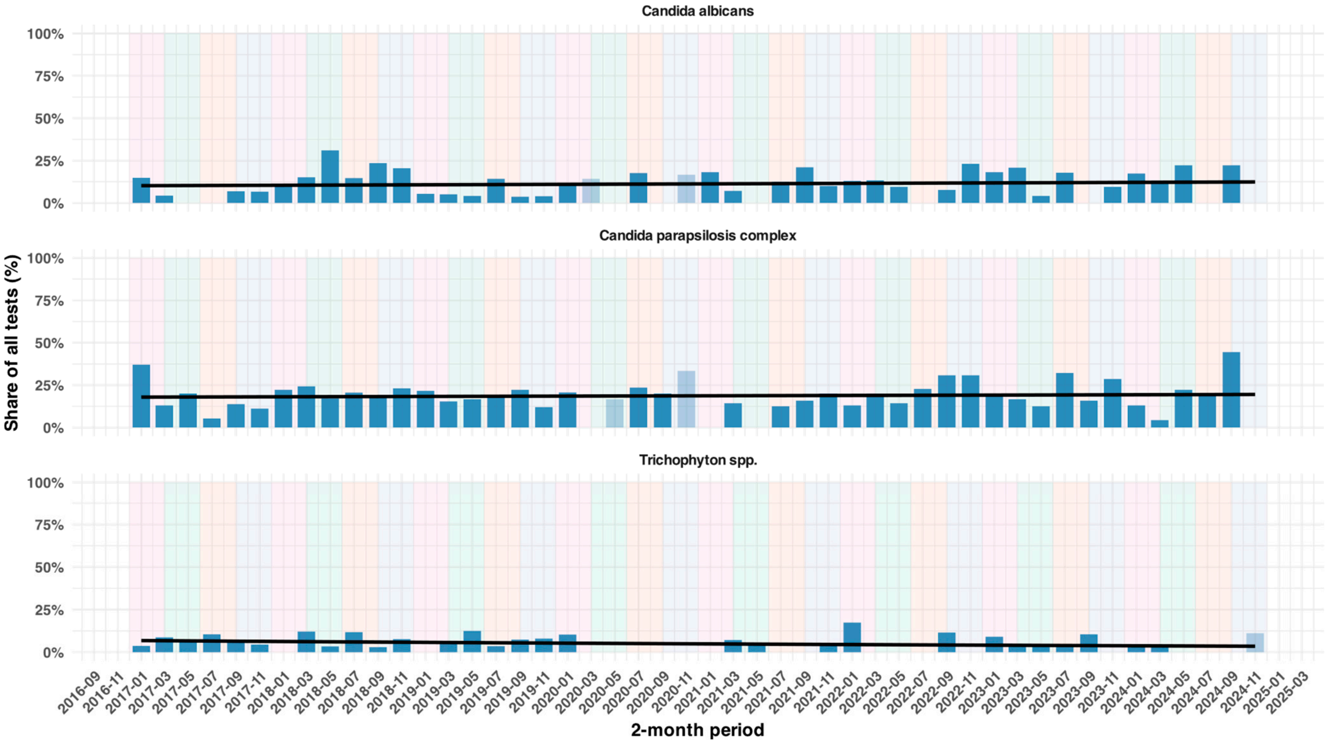

Figure S1. Bimonthly distribution of selected fungal species isolated from fingernails. Bars show the percentage of positive samples in each 2-month period, the black line represents a binomial generalized linear model (binomial GLM) fit weighted by the total number of samples, and semi-transparent bars indicate periods with fewer than 10 samples.

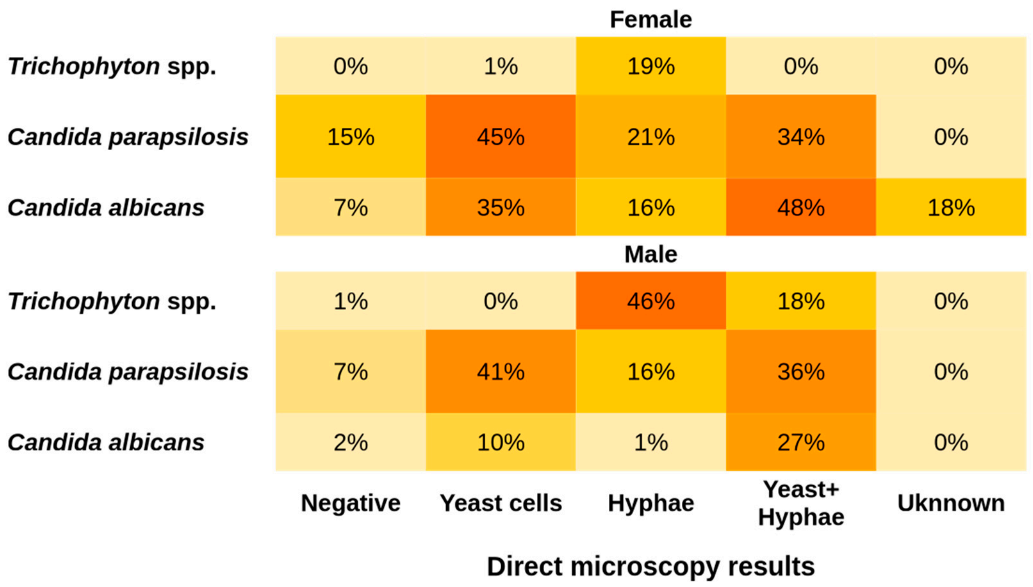

Figure S2. Heatmap showing direct microscopy findings across major fungal groups identified in culture. Percentages are based on all samples in each microscopy-by-sex group; a single sample can contribute to more than one fungal group if multiple organisms are present.

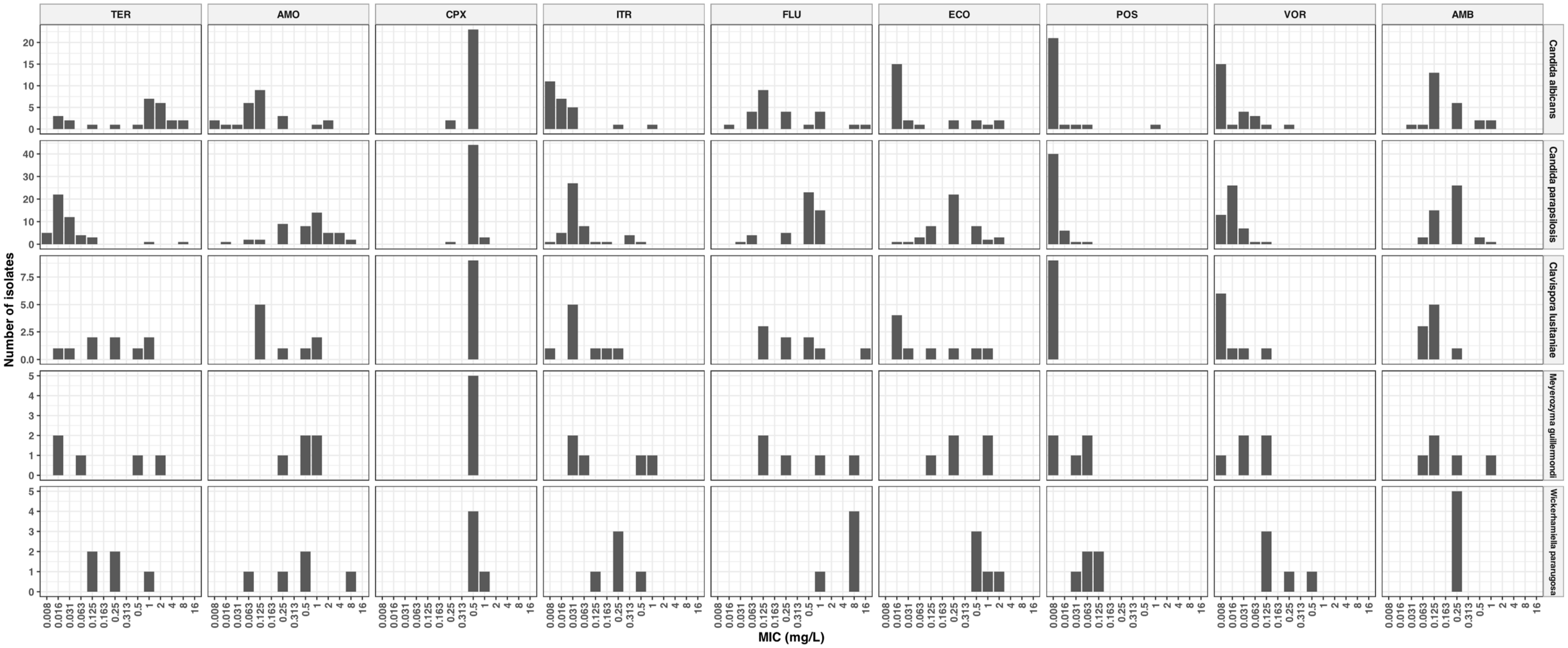

Figure S3. MIC distributions for fungal species–drug combinations with at least five isolates. Each panel shows the distribution of minimum inhibitory concentration (MIC) values for a specific species–antifungal pair. Bars represent the number of isolates at each observed MIC value (mg/L).

Table S1. Minimal inhibitory concentration (MIC) values (mg/L) for yeasts represented by small numbers in the study material.

Species (n)	AMB	AMO	CPX	ECO	FLU	ITR	POS	TER	VOR
Candida dubliniensis (1)	0.25	0.031	1	0.016	0.25	0.031	0.008	4	
Candida metapsilosis (1)	0.125	0.125	1	0.125	1	0.063	0.016	0.016	
Candida orthopsilosis (2)	0.125	1	0.5	16	64	1	0.063	0.025	8
	0.25	0.063	0.5	0.016	0.125	0.016	0.008	1	0.008
Candida sake (1)	0.063	0.25	0.5	0.5	8	0.25	0.031	0.25	0.25
Candida tropicalis (3)	0.125	0.031	0.5	0.016	0.125	0.008	0.008	0.5	0.008
	0.125	0.25	0.5	0.5	0.125	0.125	0.031	2	0.5
	0.5	0.063	0.5	0.25	1	0.031	0.016	8	0.063
Candida zeylanoides (2)	0.25	0.016	0.5	0.031	1	0.016	0.008	0.016	0.031
	0.125	0.063	0.5	0.016	0.125	0.016	0.008	1	0.008
Hanseniospora uvarum (1)	0.25	0.5	0.5	1	1	0.25	0.125	0.25	0.125
Kazachstania sanazzi (1)	0.031	0.016	0.25	0.016	0.25	0.031	0.063	0.25	0.25
Kluyveromyces marxianus (1)	0.5	0.125	0.5	0.016	0.5	0.063	0.016	8	0.031
Lodderomyces elongiosporus (2)	0.063	0.016	0.25	0.016	0.063	0.008	0.008	0.008	0.008
	0.063	0.063	0.5	0.031	0.125	0.031	0.016	0.016	0.008
Magusiomyces cepitatus (1)	1	4	0.5	2	16	0.25	0.25	2	0.25
Naganishia diffluens (2)	0.25	8	0.25	1	64	8	8	8	8
	0.25	8	0.25	1	64	8	8	8	8
Rhodothorula sp. (1)	0.25	0.25	0.25	1	64	8	8	8	2
Yarrowia lypolytica (1)	0.25	0.063	0.5	0.016	0.125	0.016	0.008	2	0.008

Table S2. Concordance between direct microscopic evaluation of fingernail samples and culture results. The table shows the number of samples by isolated fungal groups (dermatophytes, ascomycetous yeasts, basidiomycetous yeasts, non-dermatophyte moulds, culture-negative) and by microscopy findings.

Culture result	Microscopic examination result				Total
	Hyphae	Yeasts	Mixed	Negative	
**Dermatophytes**	49	1	2	4	**56**
**Ascomycetous yeasts**	35	130	29	165	**359**
**Basidiomycetoeus yeasts**	4	3	1	19	**27**
**Non dermatophyte- moulds**	14	1	1	10	**26**
**Negative**	38	18	7	532	**595**
**Total**	**140**	**153**	**40**	**730**	

Table S3. Distribution of periungual swab outcomes across operational diagnostic categories. Swab outcomes were classified as *swab negative* (no growth), *swab intermediate* (1–4 colonies), *swab positive concordant* (growth in both swab and nail samples), and *swab positive discordant* (growth detected in the swab only). Percentages are shown within each swab category across diagnostic groups (NEG, DbtPOS, PrbPOS, PssPOS, PrvPOS; see Table 1 in the main text). Swab outcomes were significantly associated with diagnostic classification (χ^2, $p < 0.001$).

Periungual swab outcome	NEG	DbtPOS	PrbPOS	PssPOS	PrvPOS	Total
**Total negative swab**	78	14	5	8	14	119
• **negative concordance (swab– / nail–)**	69 (88.5%)	10 (71.4%)	0	0	0	79 (66.4%)
• **discordant (swab– / nail+)**	9	4	5	8	14	40
**Total positive swab**	33 (29.7%)	19 (57.8%)	7 (58.3%)	19 (70.4%)	45 (76.3%)	123 (50.8%)
• **positive ≥5 CFU**	17	14	1	17	40	89
• **intermediate 1–4 CFU**	16	5	6	2	5	34
• **concordant growth (swab+, nail+)**	9	9	7	19	45	89
• **concordant species**	8 (89.9%)	8 (89.9%)	6 (85.7%)	19 (100%)	42 (93.3%)	83 (93.3%)
• **discordant (swab+, nail–)**	24	10	0	0	0	34
**Total**	**111**	**33**	**12**	**27**	**59**	**242**
• **Overall concordance (swab–/nail–) ∪ (swab+/nail+)**	77 (69.4%)	18 (54.5%)	6 (50%)	19 (70.4%)	42 (71.2%)	162 (66.9%)

Table S4 Comprehensive distribution and frequency of all fungal species identified in fingernail onychomycosis

Group of Fungi		Genus	Species	Number	Percentage
YEAST	ASCOMYCETOUS	*CANDIDA*	*Candida albicans*	120	18.52
			Candida dubliniensis	2	0.31
			Candida metapsilosis	2	0.31
			Candida orthopsilosis	6	0.93
			Candida parapsilosis	200	30.86
			Candida tropicalis	11	1.70
				341	52.62
		HANSENIOSPORA	*Hanseniospora opuntiae*	1	0.15
			Hanseniospora uvarum	10	1.54
				11	1.70
		PICHIA	*Pichia inconspicua*	1	0.15
			Pichia kudriavzevii	3	0.46

Group of Fungi		Genus	Species	Number	Percentage
				4	0.62
		OTHER SPECIES	*Clavispora lusitaniae*	27	4.17
			Cyberlindnera jadinii	1	0.15
			Debaryomyces hansenii	7	1.08
			Geotrichum candidum	8	1.23
			Kluyveromyces marxianus	1	0.15
			Kurtzmaniella zeylanoides	6	0.93
			Lodderomyces elongisporus	4	0.62
			Magnusiomyces capitatis	1	0.15
			Meyerozyma guilliermondii	13	2.00
			Nakaseomyces glabratus	1	0.15
			Saccharomyces cerevisiae	3	0.46
			Spathaspora sake	1	0.15
			Starmerella magnoliae	1	0.15
			Sungouiella intermedia	8	1.23
			Torulaspora delbrueckii	1	0.15
			Wickerhamiella pararugosa	10	1.54
			Yarrowia lipolytica	13	2.00
				106	16.36
			Candida sp. (not species ID)	23	3.55
				485	74.85
	**BASIDIOMYCETOUS**	*AUREOBASIDIUM*	*Aureobasidium pullulans*	4	0.62
		SKIN CRYTPOCOCCI	*Filobasidium, Naganishia, Papillotrema,*	24	3.7
		RHODOTORULA	*Rhodotorula mucilaginosa*	1	0.15
			Rhodotorula *sp. (not species ID)*	42	6.48
				43	6.64
		TRICHOSPORON	*Trichosporon faecale*	1	0.15
			Trichosporon japonicum	1	0.15
				2	0.30
				73	11.27
				558	86.12
**DERMATOPHYTES**		*TRICHOPHYTON*	*Trichophyton interdigitale/mentagrophytes* COMPLEX	4	0.62
			Trichophyton rubrum	51	7.87
			Trichophyton tonsurans	1	0.15
				56	8.64
**MOULDS**		*ASPERGILLUS*	*Aspergillus flavus*	1	0.15
			Aspergillus fumigatus	1	0.15
			Aspergillus niger	1	0.15
			Aspergillus versicolor	6	0.93
			Aspergillus sp.	1	0.15
				10	1.54
		FUSARIUM	*Fusarium solani*	1	0.15
			Fusarium sp. (no species ID)	12	1.85
				13	2.01
		OTHER GENRA	*Acremonium sp.*(no species ID)	2	0.31
			Alternaria sp. (no species ID)	3	0.46
			Chaetomium sp. (no species ID)	1	0.15
			Chrysosporium sp. (no species ID)	2	0.31

Group of Fungi	Genus	Species	Number	Percentage
		Scedosporium sp. (no species ID)	1	0.15
		Scopulariopsis brevicaulis	2	0.31
			11	1.70
			34	5.25
**TOTAL**			648	100
